# A genetically engineered microRNA-34a prodrug demonstrates anti-tumor activity in a canine model of osteosarcoma

**DOI:** 10.1371/journal.pone.0209941

**Published:** 2018-12-31

**Authors:** Fernando Alegre, Amanda R. Ormonde, Kellie M. Snider, Kevin Woolard, Ai-Ming Yu, Luke A. Wittenburg

**Affiliations:** 1 Department of Surgical and Radiological Sciences, School of Veterinary Medicine, University of California Davis, Davis, California, United States of America; 2 Department of Pathology, Microbiology and Immunology, School of Veterinary Medicine, University of California, Davis, Davis, California, United States of America; 3 Department of Biochemistry and Molecular Medicine, School of Medicine, University of California Davis, Sacramento, California, United States of America; Universite de Nantes, FRANCE

## Abstract

Osteosarcoma (OSA) represents the most common primary bone tumor in humans and pet dogs. Little progress has been made with regard to viable treatment options in the past three decades and patients presenting with metastatic disease continue to have a poor prognosis. Recent mouse studies have suggested that microRNA-34a (miR-34a) may have anti-tumor activities in human OSA models. Due to the conservation of microRNA across species, we hypothesized that a bioengineered miR-34a prodrug (tRNA/miR-34a) would have similar effects in canine OSA, providing a valuable preclinical model for development of this therapeutic modality. Using a panel of canine OSA cell lines, we found that tRNA/miR-34a reduced viability, clonogenic growth, and migration and invasion while increasing tumor cell apoptosis. Furthermore, canine OSA cells successfully process the tRNA/miR-34a into mature miR-34a which reduces expression of target proteins such as platelet derived growth factor receptor alpha (PDGFRα), Notch1 and vascular endothelial growth factor (VEGF). Additionally, our subcutaneous OSA xenograft model demonstrated *in vivo* tumor growth delay, increased necrosis and apoptosis by tRNA/miR-34a, and decreased cellular proliferation ability. Taken together, these data support that this novel microRNA-based therapy may possess clinical utility in a spontaneously-occurring large animal model of OSA, which can then serve to inform the clinical development of this therapy for human OSA patients.

## Introduction

Osteosarcoma (OSA) is an aggressive, high-grade bone neoplasm derived from mesenchymal stem cells with the capacity to produce osteoid and represents the most common primary malignant tumor of bone in humans and dogs [1, 2]. Human and canine OSA share notable clinical, histological and molecular similarities, such as predilection for metaphysis of weight-bearing bones, metastatic pattern (lungs, bones, lymph nodes), undetectable pulmonary micro-metastasis at the time of diagnosis, multimodal therapy, and dysregulation of several critical molecular pathways (e.g. P53, PTEN, RB, MYC), among others [3, 4]. Furthermore, genetic analyses of canine and human OSA have revealed a high degree of genomic reorganization with shared conserved genomic alterations between species and remarkably similar gene expression signatures [5, 6]. Despite these similarities, canine OSA is more prevalent with an occurrence at least 10-fold higher than humans, with around 10,000 new cases diagnosed annually compared to 800 cases diagnosed in humans [1, 2]. Therefore, canine OSA represents a valuable tool in which to study novel treatment strategies. Indeed, dogs have been employed as naturally occurring cancer model of human OSA not only to study the biology of the disease but also to evaluate pioneering therapies that are now used in humans, including limb salvage techniques and immunotherapy treatments encapsulated in liposomes [7, 8]. Although the current multidrug therapy combined with aggressive surgical techniques have improved survival in both species, the 5-year survival remains halted about 60–70% in humans and 10–20% in dogs over the last 50 years [4, 9]. Therefore, there is a desperate need for developing novel forms of therapy to improve upon the currently achievable clinical results in OSA.

MicroRNAs (miRNA or miR) comprise a highly conserved and large family of small non-coding RNAs (18–22 nucleotides) that play a critical role in the post-transcriptional regulation of protein-coding genes [10, 11]. There is substantial evidence that miRNA expression is dysregulated in cancer compared to normal cells and they can have either tumor-suppressive or oncogenic functions, by targeting genes implicated in hallmarks of cancer, such as evasion of growth suppressors, resistance to cell death, activation of invasive phenotype and induction of angiogenesis [11, 12]. These relevant features, besides the ability to target several mRNA altered in cancer, makes miRNAs stimulating candidates as novel therapeutic targets in cancer [13, 14] While their role in osteosarcoma is still being deciphered, miRNA dysregulation has been correlated with aggressive behavior, chemoresistance, shorter survival time and enhance lung metastasis in both canine and human OSA [15, 16]. Moreover, both species share a comparable miRNA signature in OSA disease [3, 15, 17], reinforcing canine OSA as a model to study novel cancer therapies. Among the miRNAs involved in OSA development and progression is miR-34a, whose expression is significantly downregulated in both canine and human OSA [18, 19]. Lower miR-34a expression in tumor and plasma has been associated with a shorter disease-free survival time, poor prognosis [20] and chemoresistance [21]. miR34-a, transcriptionally regulated by p53, acts as tumor suppressor through the regulation of multiples genes involved in tumorigenesis and cancer progression, such as *NOTCH1*, platelet-derived growth factor receptor alpha (*PDGFRA*), NAD-dependent deacetylase sirtuin-1 (*SIRT1*) and *SURVIVIN*, as well as p53 target genes [22–25]. Indeed, the restoration of miR-34a levels in OSA cells through administration of synthetic miR-34a or miR-34a-expressing vectors, has confirmed the anti-tumor effects of this miRNA which induces apoptosis and G_1_-G_2_ cell cycle arrest, inhibits cell proliferation, migration and invasion as well as cell adhesion, and leads to microtubule cytoskeleton destabilization in OSA cells. Additionally, orthotopic mouse models treated with chemically synthesized miR-34a-mimics attenuated tumor growth, pulmonary metastasis, and enhanced the sensitivity of malignant cells to chemotherapeutic drugs [19, 25–28]. Given the extensive amount of oncogenic mRNA that are regulated by miR-34a and its relevant effects in cancer cells, there has been an effort to develop successful miR-34a-based therapies [13].

Recently, Yu *et al* have developed a novel method to synthesize large quantities of a bio-engineered miR-34a prodrug [29]. This novel miR-34a prodrug (a tRNA/miR-34a chimera) lacks many of the chemical modifications contained in synthetic miRNA and is selectively processed within human tumor cells to mature, fully functional miRNA that is capable of reducing protein levels of its target genes and consequently suppressing human OSA cell proliferation. Furthermore, systemic and intra-tumoral administration of this bioengineered miR-34a prodrug in an orthotopic human OSA xenograft mouse successfully attenuated tumor growth associated with increased tissue necrosis and without presence of liver or kidney toxicity [29, 30]. Taking into account that miR-34 is highly conserved across species and canine OSA is a well-known, naturally occurring model of human OSA, we aimed to evaluate the effectiveness of this human bioengineered miR-34a prodrug in canine OSA cells in order to validate canine OSA as a useful model for informing future clinical development of this novel therapeutic agent.

## Materials and methods

### Cell lines and conditions

Four canine OS cell lines were used in this study: HMPOS, Abrams, Gracie (*p53 mutant*) and D-17 (*p53 wild-type*) [31–33]. Abrams, Gracie and HMPOS were kindly provided by Dr. Dawn Duval (Department of Clinical Sciences and Flint Animal Cancer Center at Colorado State University, Fort Collins, CO) [17, 34–36]. The D-17 cell line was obtained from ATCC (Cat# CCL-183, Manassas, VA). These cell lines were previously validated and characterized at Colorado State University [17], and were verified to be free from mycoplasma contamination by our laboratory. Cells were serially passaged by trypsinization in RPMI 1640 culture medium (Mediatech Inc., Manassas, VA) supplemented with 10% heat-inactivated fetal bovine serum (Invitrogen, Carlsbad, CA) and 50 U/mL penicillin 50 μg/mL streptomycin (Mediatech Inc). For experimental procedures, cells were incubated at 37°C in a humidified environment with 5% CO_2_.

### Genetically engineered chimeric miR-34a prodrug

The chimeric recombinant tRNA fusion pre-miR-34a (sephadex aptamer tagged methionyl tRNA fusion pre-mir-34a or tRNA/miR-34a) and the control scaffold (sephadex aptamer tagged methionyl tRNA or tRNA/MSA) were biosynthesized as previously described in the laboratory of Dr. Aiming Yu [37]. The purity of recombinant tRNA/miR34-a prodrug was evaluated by denaturing urea (8 M) polyacrylamide gel (8%) electrophoresis and high-performance liquid chromatography (HPLC) assays. Highly purified tRNA/miR-34a (> 98% purity) was employed in this research.

### Cell viability and proliferation

#### MTT assay

Cell viability and proliferation of the canine OS cell lines (Abrams, HMPOS, GD-17 and Gracie) treated with tRNA/miR34-a was determined using a MTT (3-(4,5-Dimethylthiazol-2-yl)-2,5-Diphenyltetrazolium Bromide) based-colorimetric assay (TACS MTT Cell Proliferation Assay, Trevigen, Gaithersburg, MD), according to manufacturer’s instructions. Briefly, canine OS cells were seeded in 96-well cell culture microplates (Corning Costar, Corning, NY). After 24 hours, cells were treated with tRNA/miR-34a or tRNA/MSA (0, 1, 5, 10, 20 and 30 nM) in triplicate by means of jetPRIME transfection reagent (Polyplus-transfection SA, New York, NY). After 8 hours, transfection mixture was replaced with supplemented RPMI 1640 medium. MTT assays were performed 48 hours after treatment. Cells were incubated with MTT reagent in the dark at 37°C for 2 hours. The detergent reagent was then added to all wells, and plates were returned to the dark at 37°C for other additional 2 hours. Following the incubation, absorbance was measured at 570 and 690 nm employing a microplate spectrophotometer (SpectraMax 190, Molecular Devices LLC., Sunnyvale, CA). IC_50_ and Hill Slope values were calculated using the non-linear regression inhibitory concentration versus normalized analysis with variable slope implemented in GraphPad Prism v7.04 (GraphPad Software, La Jolla, CA).

#### Resazurin assay

The time-dependent anti-proliferative effects of tRNA/miR-34a prodrug on the canine OSA cells were evaluated by means of the resazurin-based fluorometric assay (R7017, Sigma-Aldrich, St. Louis, MO). To perform this assay, cells were seeded in black 96-well plates (Corning Costar) at different densities depending on the length of the treatment and allowed to attach overnight. Cells were then treated with 5 nM tRNA/miR-34a, tRNA/MSA or vehicle in triplicate for 24, 48, 72 or 96 hours. Following the treatment, culture medium was aspirated and cells were incubated with 10% resazurin in supplemented culture medium for 2 hours. Fluorescence (excitation 530 nm and emission 590 nm) was then measured using a microplate reader (Synergy H1, BioTek, Winooski, VT).

#### Clonogenic assay

D-17 and HMPOS cells were treated with 5 nM tRNA/miR-34a or tRNA/MSA, or vehicle for 24 hours, and subsequently cells were detached by trypsinization, counted and seeded in a 6-well plate (Corning Costar) in triplicate at 300 cells per well in 2 mL supplemented RPMI 1640 medium. After 7 days of incubation, colonies were gently rinsed with warm PBS and then fixed with 95% ethanol:acetic acid solution (5 min), stained with crystal violet (10 min) and manually counted.

### Apoptosis analysis

#### Caspase-3/-7 activity

Measurement of the enzymatic activities of caspase-3/-7 was carried out with a fluorometric SensoLyte Homogeneous AMC Caspase-3/-7 Assay Kit (AnaSpec, Fremont, CA), in accordance with manufacturer’s recommendations. Briefly, canine OSA cells were exposed to tRNA/miR-34a or tRNA/MSA, or vehicle for 48 hours in a black 96-well microplate (Corning Costar). After treatment, 50 μL/well of caspase-3/-7 substrate solution was added into each well, and the microplate was incubated at room temperature (RT) for 1 hour in the dark. Fluorescence intensity was evaluated at 354 nm/442 nm (excitation/emission) on a fluorescence microplate reader (Synergy H1, BioTek).

#### TUNEL assay

Canine OSA cells were cultured on 4-well, chambered culture slides (Falcon CultureSlides, Corning) and treated with 5 nM tRNA/miR-34a or tRNA/MSA, or vehicle for 48 hours. Subsequently, cells were washed with PBS and allowed to air dry. Cells were fixed (4% paraformaldehyde in PBS, pH 7.4) for 1 hour, at RT. Following a washing step with PBS, cells were incubated in permeabilisation solution (0.1% Triton X-100 in 0.1% sodium citrate) for 2 min on ice and washed again. Finally, slides were incubated with TUNEL (Terminal deoxynucleotidyl transferase dUTP nick end labeling) reaction mixture in a humidified atmosphere for 1 hour at 37°C, washed three times with PBS and mounted with VectaShield plus DAPI mounting medium (H1500, Vector Laboratories Inc, Burlingame, CA). TUNEL assay was also performed in cryopreserved xenograft tumor sections; TUNEL staining was carried out after heat induced epitope retrieval employing 0.1 M Citrate buffer (pH 6.0) and blocking non-specific binding sites using Tris-HCl 0.1 M, pH 7.5, containing 3% bovine serum albumin (BSA) and 20% normal goat serum. Ten random fields (20x) were acquired with a Leica EL6000 attached to a Leica DMI3000B inverted microscope (Leica Mycrosystems, Buffalo Grove, IL).

#### Bivariate Annexin V/Propidium Iodide analysis

Following 48 hours treatment with tRNA/miR-34a, tRNA/MSA or vehicle, cells were collected, washed with 1X Binding Buffer twice and stained employing the eBioscience Annexin-V Apoptosis Detection Kit FITC (Invitrogen, Carlsbad, CA) according the manufacturer’s instructions. Stained cells were analyzed on a CytoFLEX S flow cytometer (Beckman Coulter, Indianapolis, IN) and data were processed using FlowJo v4.0 software (FlowJo LLC, Ashland, OR).

### Cell migration and invasion assay

To assess the invasive behavior of canine OS cells exposed to tRNA/miR-34a prodrug we employed 24-well Corning BioCoat Matrigel Invasion Chamber (Corning). HMPOS and D-17 cells treated with 5 nM tRNA/miR-34a or tRNA/MSA for 48 h were harvested and counted, and a cell suspension of 4.5 x 10^4^ (HMPOS) or 1.0 x 10^5^(D-17) cells in serum-free media was added to the upper matrigel-coated insert. Media containing 10% FBS was added to lower chamber and incubated at 37°C for 20 hours. After removing non-invading cells from the upper surface of the insert, those on the lower surface were fixed in 95% ethanol:acetic acid solution and stained with crystal violet. Invading cells were photographed (seven random 20x fields) and counted under a Leica DM2000 phase-contrast inverted microscope (Leica Mycrosystems). Cells were treated in duplicate and assayed separately. Invasion index was determined by dividing % invasion of treated cells by the one of control cells. Migration was evaluated in parallel, following the same procedure above using the Corning BioCoat Control Inserts with 8.0μm pores instead of matrigel-coated inserts.

### Quantitative RT-PCR

Quantitative RT-PCR was performed using mRNA extracted from canine OS cells treated with tRNA/miR-34a or tRNA/MSA (5–10 nM), or vehicle. Cells were collected after 24–48 hours post-treatment and total RNA was isolated using TRIzol Reagent (Invitrogen), according to manufacturer’s directions. cDNA was synthetized using a M-MuLV Reverse transcriptase (New England Biolabs, Ipswich, MA) and a RT-primer 5′-CAGGTCCAGTTTTTTTTTTTTTTTVN (where V is A, C, and G and N is A, C, G, and T) (Sigma-Aldrich) for miR-34a as described elsewhere [38], or a Quantitec Reverse Transcription Kit (Qiagen Inc., Germantown, MD) for *Rps5*, *Gapdh*, *hnRnph1*, *Pdgfra*, *Notch1*, *Sirt1* and *Survivin*. qRT-PCR reactions (in duplicate) were performed employing a QuantiFast SYBR Green PCR Kit (Qiagen) on a Rotor-Gene Q real time PCR cycler (Qiagen). Sequences of canine gene primers are shown in Table 1. The geometric mean of housekeeping genes Rps5, Gapdh and *Hnrnph1* was used to normalize the expression of genes of interest. The threshold cycle (C_T_) was determined and relative gene expression was calculated using the comparative Ct method 2^−Δ(ΔCT)^ [39].

**Table 1:** Primer sets of canine genes used for qRT-PCR.

Gene Symbol	Sequence direction	Sequence (5’→3’)
*Pdgfra*	sense	CTCACTTATCGTCCTGGTTGTC
	antisense	GCATCGGGTCCACGTAAATA
*Notch1*	sense	GTGGCTATTCCTGTGAGTGTGT
	antisense	GTAGGTGTTGATGAGGTCGATG
*Sirt1*	sense	CAGTTCCAACCATCTCTCTGTC
	antisense	GCAACCTGTTCCAGTGTATCT
*Rps5* [40]	sense	TCACTGGTGAGAACCCCCT
	antisense	CCTGATTCACACGGCGTAG
*Survivin* [41]	sense	TCGAAGAGACCGCAAAGAAAGTGC
	antisense	GAATTGTGGCCGTTCTCCTTTCCT
*Hnrnph1* [42]	sense	CTCACTATGATCCACCACG
	antisense	TAGCCTCCATAACCTCCAC
*Gapdh*	sense	AACATCATCCCTGCTTCCAC
	antisense	AGACCACCTGGTCCTCAGTG
*Mir34a*	sense	GCAGTGGCAGTGTCTTAG
	antisense	GGTCCAGTTTTTTTTTTTTTTTACAAC

### Protein extraction and western blot analysis

Cells were treated with tRNA/miR-34a or tRNA/MSA (5 nM, 48 hours) or vehicle, and were collected in ice-cold PBS and centrifuged. Whole-protein extracts were obtained by lysing cell pellets with RIPA Lysis Buffer [Tris-HCl 50 mM, 150 mM NaCl, 5 mM EDTA, 0.1% Triton X-100, 0.5% sodium deoxycholate, 0.1% SDS and supplemented with cOmplete Protease Inhibitor Cocktail (Roche)]. Protein concentrations were determined by bicinchoninic acid-based assay (Pierce BCA Protein Assay Kit, ThermoFisher Scientific, Waltham, MA). Whole-protein extracts (25 μg) were resolved by Tris-Acetate (7%) or Tris-Glycine (10%) and transferred to polyvinylidene difluoride membranes using a Mini Gel/Blot System (Invitrogen). After blocking with 5% non-fat milk or bovine serum albumin, membranes were incubated with primary antibodies: anti-PDGFRa (#3174), anti-Notch1 (#3608) (Cell Signaling, Boston, MA) or anti-ß-actin (MA1-91399, ThermoFisher Scientific); and horseradish peroxidase-conjugated secondary anti-mouse (#32430) or anti-rabbit (#32260) antibodies (Invitrogen). The chemiluminescence signal was detected using Luminata Forte Western HRP substrate (Millipore, Billerica, MA), SuperSignal West Pico or SuperSignal West Femto (both from Thermo Scientific) and was visualized with a digital image analyzer (FluorChem E, ProteinSimple, San Jose, CA).

### VEGF measurement

VEGF concentration in cell supernatants was determined employing a commercially available enzyme-linked immunosorbent assay (ELISA) kit (Human VEGF Quantikine ELISA Kit, #DVE00, R&D Systems, Minneapolis, MN), according to the manufacturer’s instructions. Cells were plated in 24-well microplates and transfected with 5 nM tRNA/MSA or tRNA/miR-34a, or vehicle and incubated for 24, 48 or 72 hours. All the treatments were performed in duplicate. Following incubation, cell supernatants were collected and centrifuged at 500×g to remove any particles or cellular debris prior to performing the assay. Optical density was determined at 450 nm using a Synergy H1 (BioTek) microplate reader.

### Animal study

Animal experiments were performed under and Institutional Animal Care and Use Committee-approved protocol (#19716) and institutional guidelines for animal welfare in an AALAC-accredited facility. Twenty four NCr-nu/nu athymic mice (4-to-6 week old) were purchased from the Charles River Frederick Research Model Facility (Frederick, MD) and housed in ventilated caging. After a one-week acclimatization period, Abrams canine OS cells (2 x 10^6^ in 0.9% NaCl) were injected subcutaneously on the right flank. Seven days after tumor inoculation, tumors were size-matched, divided into three groups and treated in the following cohorts: tRNA/miR34a prodrug (nine mice) formulated in *in vivo*-JetPEI (Polyplus-transfection SA) according to the manufacturer instructions, tRNA/MSA (nine mice) formulated in *in vivo*-JetPEI and vehicle control group (six mice) receiving only *in vivo*-JetPEI. Mice received treatments via tail vein injection (100 μL) consisting of a 50 μg initial loading dose on Day 1 followed by 25 μg maintenance doses three times per week for 5 doses. Three mice from the tRNA/miR-34a group and three mice from the tRNA/MSA control group (randomly selected prior to initiation of treatments) were sacrificed 24 hours after the last dose and tumors were collected for evaluation of early pharmacodynamic effects. Tumor growth (six mice per group) was monitored every other day by measuring three dimensions with a caliper. Tumor volumes were calculated as *V* (*mm*3) = (*π*/6) *x L x W x H*. Animals were sacrificed when tumors reached a maximum-allowed size or morbidity due to tumor ulceration occurred (generally 22–27 days following initial treatment). Tumors were removed upon sacrifice and flash frozen in liquid nitrogen. Tumors utilized for pathologic analysis of early PD effects were cut in half upon removal; one-half was placed in 0.9% sodium chloride and then were fixed and processed for cryosectioning and analysis of apoptosis (TUNEL), cell proliferation (Ki-67 labeling) and hematoxylin-eosin (H&E) histology. The other half of the tumor was flash-frozen in liquid nitrogen.

### Histopathology

For histopathology evaluation, xenograft tumor tissues were perfused with 4% paraformaldehyde, and then fixed overnight before cryoprotection using 30% sucrose. Tissue sections were then OCT (optimal cutting temperature compound)-embedded and sectioned 20 times at 5-μm thickness, with 50-μm steps between sections 5, 10, and 15. Thus, a total of approximately 250-μm of tissue was cut into slides. 5-μm thickness sections were air dried and then stained for routine H&E evaluation with Richard-Allan Scientific Hematoxylin 7211 (ThermoFisher Scientific, Waltham, MA) and 3% Eosin Y (ACROS Organics, ThermoFisher Scientific) using an autostainer (Thermo Shandon Varistain Gemini ES, ThermoFisher Scientific).

### Ki-67 labeling

To evaluate the proliferative indices of xenograft tumors, we evaluated the nuclear protein Ki-67/MIB-1 that it is expressed in all active phases of the cell cycle but is absent in quiescent cells. Slides were treated post-fixation with ice-cold 70% methanol, blocked for 1 hour in 5% normal goat serum (SouthernBiotech, Birmingham, AL) and hybridized with a mouse monoclonal anti-MIB1 antibody (Dako, Agilent Technologies, Carpinteria, CA) for 1 hour at RT, followed by goat anti-mouse Alexa Fluor 488 fluorescent antibody (Invitrogen). Pictures were taken with a Leica EL6000 attached to a Leica DMI3000B inverted microscope (Leica Mycrosystems).

### Statistical analysis

Animal numbers were determined by power calculation using freely available software (G*Power V.3.0) with input parameters of 1) effect size of 1.942, 2) error probability of 0.05, 3) power of 0.8 and 4) two-tailed evaluation with equal allocation between treatment groups. The effect size was estimated by analysis of previously published results of an orthotopic murine model of human osteosarcoma treated similarly with the tRNA/miR-34a prodrug [43].

Comparisons of the anti-proliferative effect of tRNA/miR-34a and tRNA/MSA were compared by ANOVA with a Bonferroni multiple-comparison post-test. IC_50_ values and Hill slopes were calculated by non-linear regression of inhibitor concentration versus normalized anti-proliferative response with variable slope. Comparisons of the effect on clonogenic cell growth, caspase-3/-7 activity, TUNEL staining and VEGF ELISA were performed by two-way ANOVA with Bonferroni multiple-comparison post-test. The comparisons between the effect on tumor cell migration and invasion were performed by ordinary one-way ANOVA with Tukey’s multiple comparison test. Unpaired Student’s *t*-test were used to compare the results obtained by RT-qPCR. Comparison of tumor volumes among treatment groups in the xenograft study was done by repeated-measures two-way ANOVA followed by Bonferroni multiple-comparison post-test to evaluate differences at individual time points. Survival curves, with tumor diameter reaching 400 mm^3^ as the endpoint, were generated by the Kaplan-Meier method and survival differences between groups compared using log-rank (Mantel-Cox) analysis.

Statistical analysis was performed using GraphPad Prism v7.04. For all comparisons, a P-value ≤0.05 was considered significant.

## Results

### Canine osteosarcoma cell proliferation and clonogenic cell growth are suppressed by bioengineered miR-34a prodrug

**Fig 1:**
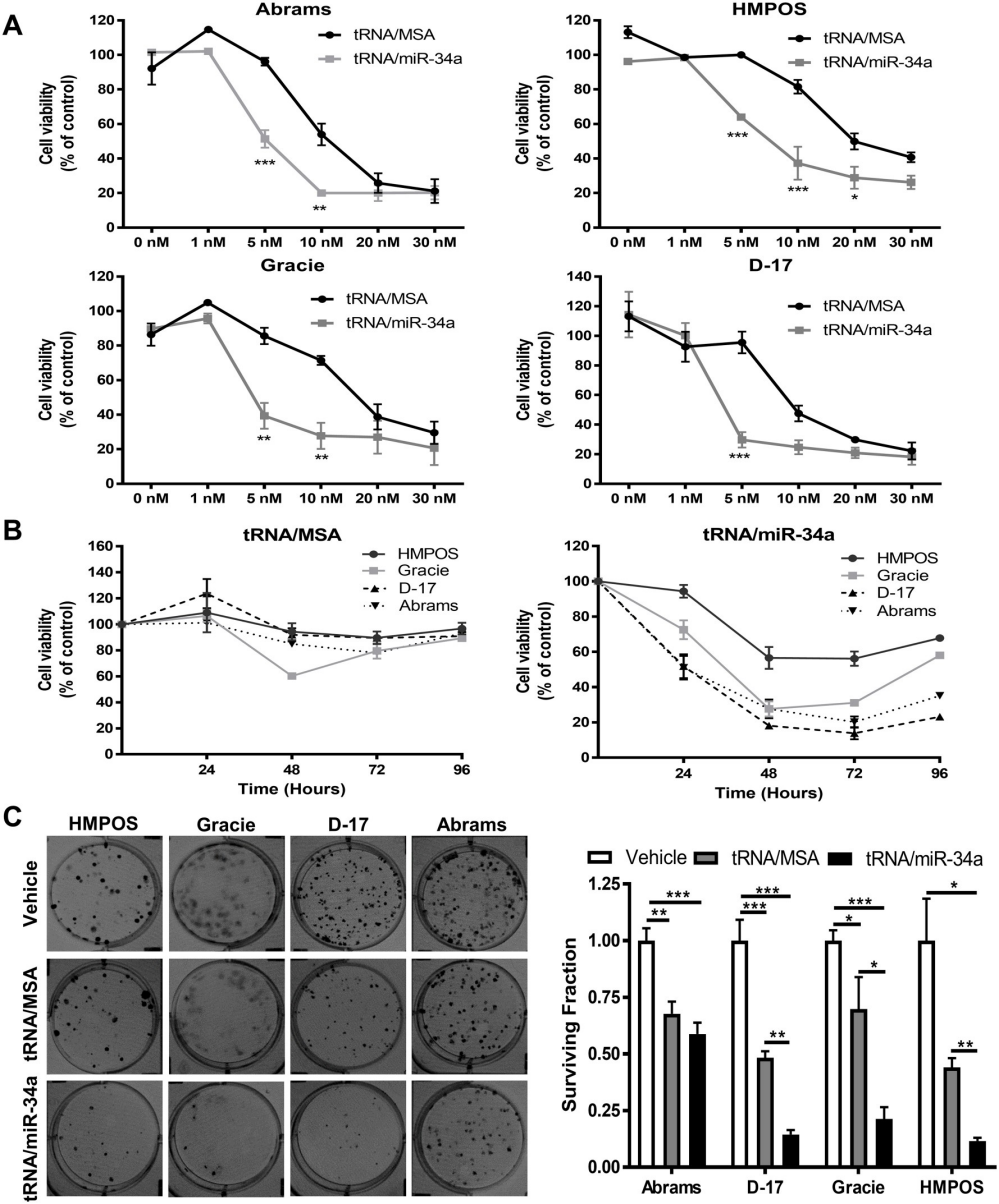
Bioengineered tRNA/miR-34a prodrug suppressed cell proliferation and clonogenic growth in canine OSA cell lines. MTT assay was employed to evaluate the cellular viability in cells transfected with (A) several concentrations (0–30 nM) of tRNA/ miR-34a or tRNA/MSA after 48 hours, and (B) cells treated with 5 nM tRNA/miR-34a or tRNA/MSA after different times post-transfection (0–96 hours). (C) Representative images and surviving fraction from the clonogenic assays. Cells treated with vehicle, tRNA/MSA or tRNA/miR-34a were plated in 6-well plates and allowed to growth into colonies for 7 days. Data (mean±SEM) were calculated as percentage of control (untreated cells) and analyzed by one-way or two-way ANOVA multiple test followed by a Bonferroni test (*p <0.05, **p <0.01, ***p <0.001).

We analyzed the anti-proliferative effects of tRNA/miR-34a prodrug using the MTT assay in Abrams, HMPOS, Gracie and D-17 cells at 48 hours following transfection. The results showed that tRNA/miR-34a prodrug significantly (P<0.001) inhibited canine OS cell proliferation in a concentration-dependent manner compared to the negative control tRNA/MSA. The latter also reduced cellular viability although to a smaller degree than the miR-34a prodrug (Fig 1A). The calculated IC_50_ and Hill Slope values for tRNA/miR-34a and tRNA/MSA are shown in Table 2. Given these findings, we performed the majority of the remaining *in vitro* experiments with both compounds at 5 nM as this concentration resulted in significant anti-proliferative effect only in the miR-34a prodrug and not control tRNA/MSA. In order to identify time-dependent anti-proliferative effects, cellular viability was evaluated by resazurin assay at 24, 48, 72 and 96 hours after transfection (Fig 1B). Our findings reveal a decrease in cellular viability in a time-dependent manner up to 72 hours with slight recovery after 96 hours. Taken together, the cell proliferation assays demonstrate that HMPOS cells appear to be the least sensitive to tRNA/miR-34a prodrug and the D-17 cells the most sensitive. Proliferation results were further confirmed by clonogenic assay. We found that both colony formation and surviving fraction in all canine OS cell lines treated with tRNA/miR-34a for 24 hours were significantly reduced (P<0.01) when compared to tRNA/MSA (Fig 1C).

**Table 2:** Estimated IC50 values for cell proliferation inhibition.

	Abrams		HMPOS		Gracie		D-17	
	tRNA/MSA	tRNA/miR-34a	tRNA/MSA	tRNA/miR-34a	tRNA/MSA	tRNA/miR-34a	tRNA/MSA	tRNA/miR-34a
IC50 (nM)	12.43±1.42	5.45±0.79	22.3±1.81	8.36±1.03	16.42±1.64	4.95±1.14	12.1±1.67	4.11±1.10
HillSlope	2.12±0.47	1.42±0.27	1.87±0.35	1.11±0.17	1.64±0.30	1.04±0.24	1.81±0.44	1.29±0.38

#### Bioengineered tRNA/miR-34a prodrug induces apoptosis in canine osteosarcoma cells

**Fig 2:**
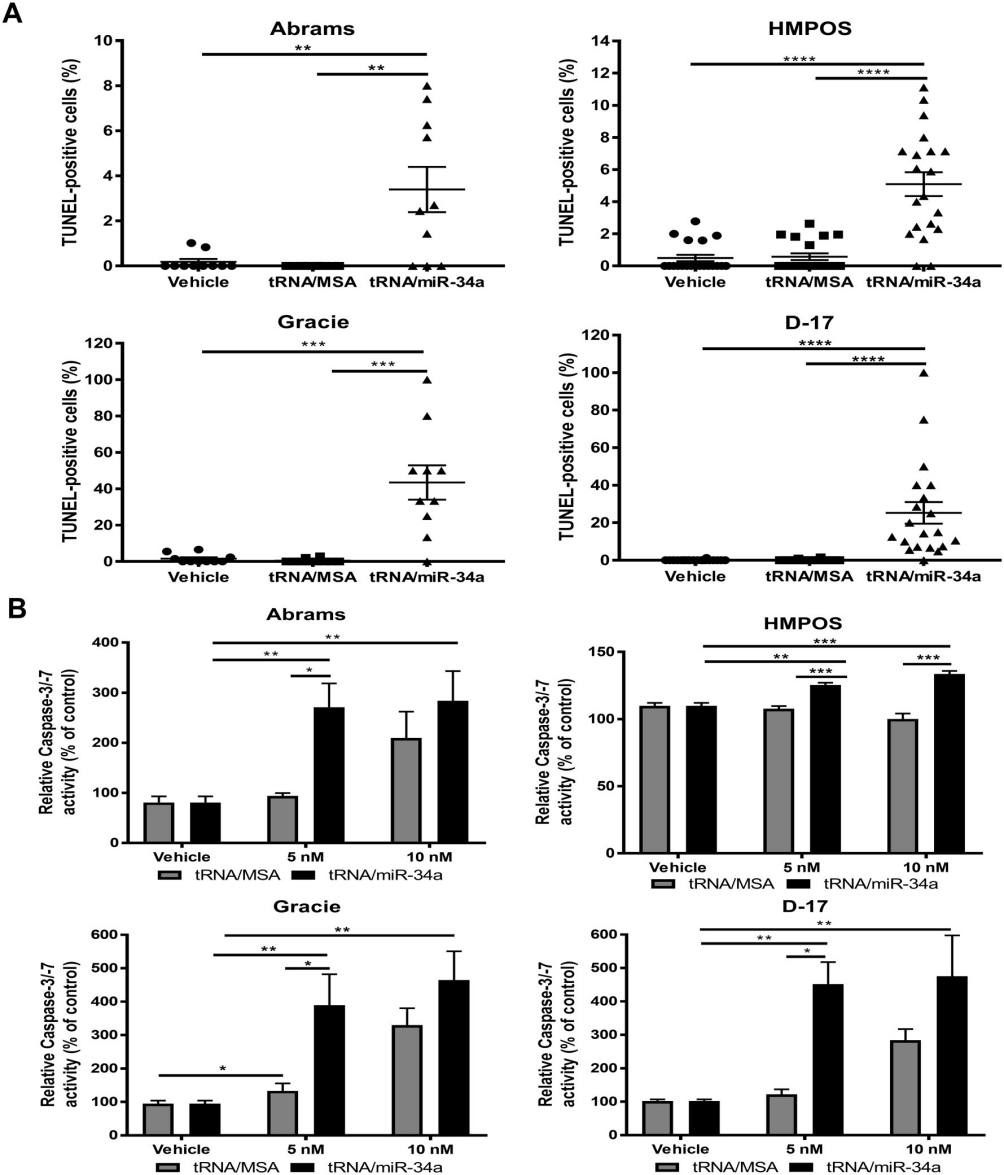
Apoptosis was enhanced in canine OSA cell lines treated with genetically engineered tRNA/miR-34a prodrug. Apoptosis was assessed by (A) TUNEL staining and (B) Caspase-3/-7 activity in cells treated with vehicle, tRNA/MSA or tRNA/miR-34a (5–10 nM) after 48 hours post-treatment. Data (mean±SEM) were calculated as percentage of (A) total number of cell, (B) control (untreated cells) and analyzed by one-way or two-way ANOVA multiple test followed by a Bonferroni test (*p <0.05, **p <0.01, ***p <0.001, ****p <0.0001).

**Fig 3:**
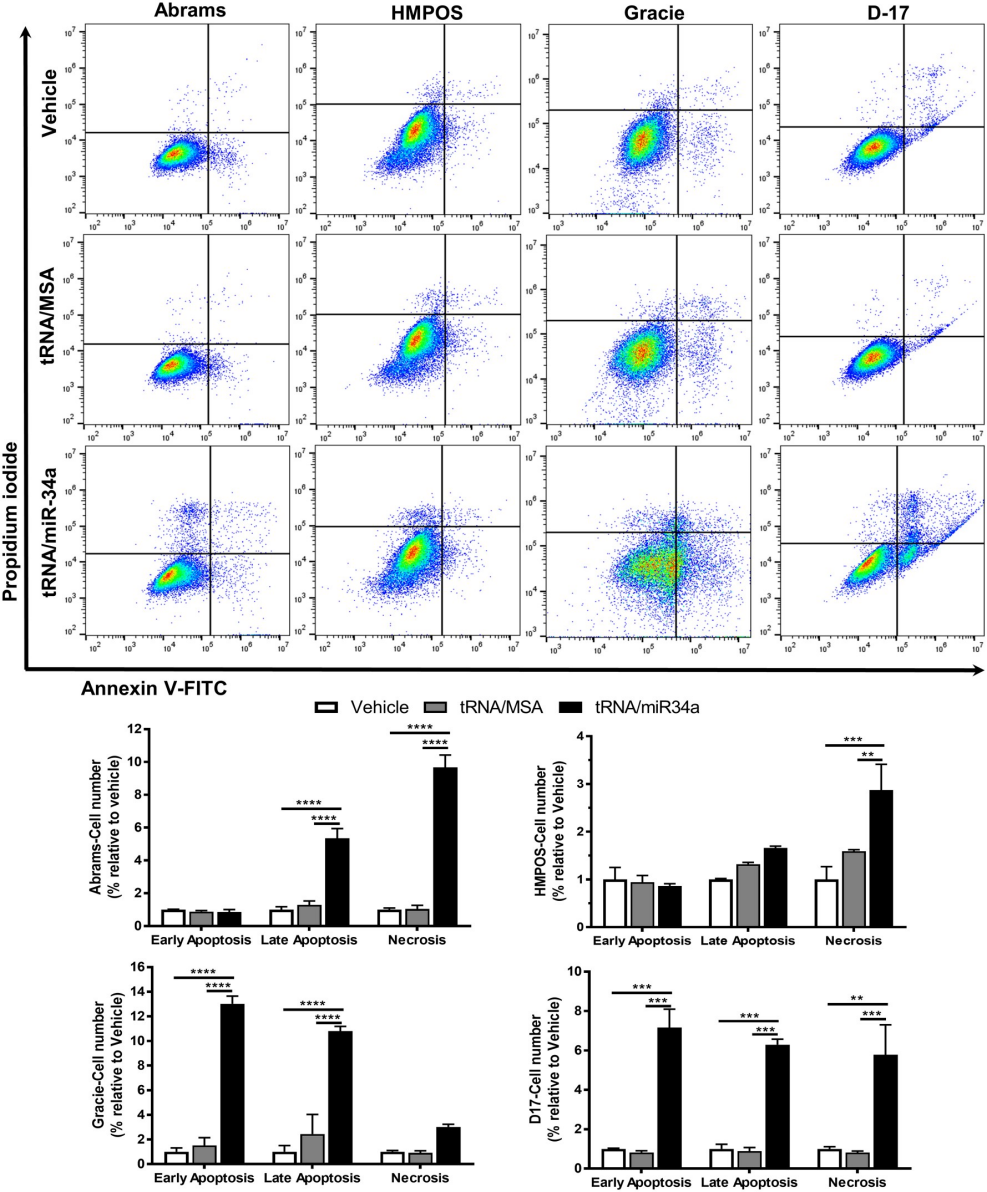
Necrosis and late apoptosis was induced in canine OSA cell lines treated with bioengineered tRNA/miR-34a prodrug. Bivariate PI/Annexin-V analysis was performed in cells treated with vehicle, tRNA/MSA or tRNA/miR-34a (5 nM) after 48 hours post-treatment. Data (mean±SEM) were calculated as percentage of control (untreated cells) and analyzed by two-way ANOVA multiple test followed by a Bonferroni test (**p <0.01, ***p <0.001, ****p <0.0001).

The proliferation assays based on bioreductive methodology that we applied do not distinguish between cell death and growth arrest. In order to ascertain whether apoptotic pathways are involved in the suppression of canine OS cell line proliferation and viability by tRNA/miR-34a prodrug, we employed a TUNEL assay evaluating DNA degradation, a feature of late apoptosis and necrosis (Fig 2A). tRNA/miR-34a treatment (5 nM) led to a significant increase in percentage of TUNEL-positive cells after 48 hours in all canine OS cell lines (P<0.001). In contrast, neither tRNA/MSA treatment nor vehicle resulted in significant increases in DNA fragmentation. Furthermore, we analyzed the activation of caspase-3/-7 using a fluorometric assay in canine OS cells treated with tRNA/MSA or tRNA/miR-34a (5–10 nM) or vehicle after 48 hours (Fig 2B). Consistent with the results of the TUNEL assay, tRNA/miR-34a prodrug significantly enhanced the caspase-3/-7 activity in a concentration-dependent manner as compared to tRNA/MSA or vehicle (P<0.01). Increased activation of caspase-3/-7 activity in the Abrams, Gracie and D-17 cell lines was seen with the tRNA/MSA treatment, although to a lower degree than the miR-34a prodrug. Although TUNEL staining and caspase-3/-7 activity assay suggest a trend towards apoptosis, additionally we evaluated the apoptosis profiles in canine OSA cells at 48 hours post-treatment (5 nM) employing a bivariate Propidium Iodide (PI)/Annexin-V analysis, which was performed by flow cytometry (Fig 3). Our results showed that tRNA/miR-34a-treated canine OSA cells displayed both late apoptosis (PI^+^/Annexin-V^+^) and necrosis (PI^+^/Annexin-V^-^) features (P<0.01). Moreover, tRNA/miR-34a significantly enhanced early apoptosis (PI^-^/Annexin-V^+^) in Gracie and D-17 cells (P<0.001). In contrast, tRNA/MSA did not trigger significantly neither apoptosis nor necrosis in canine OSA cells. Taken together, these results indicate that the reductions in cell proliferation caused by tRNA/miR-34a are, at least in part, the result in an increase in treatment-induced apoptosis.

### *In vitro* metastatic ability of canine OS cells is inhibited following transfection with bioengineered tRNA/miR-34a prodrug

**Fig 4:**
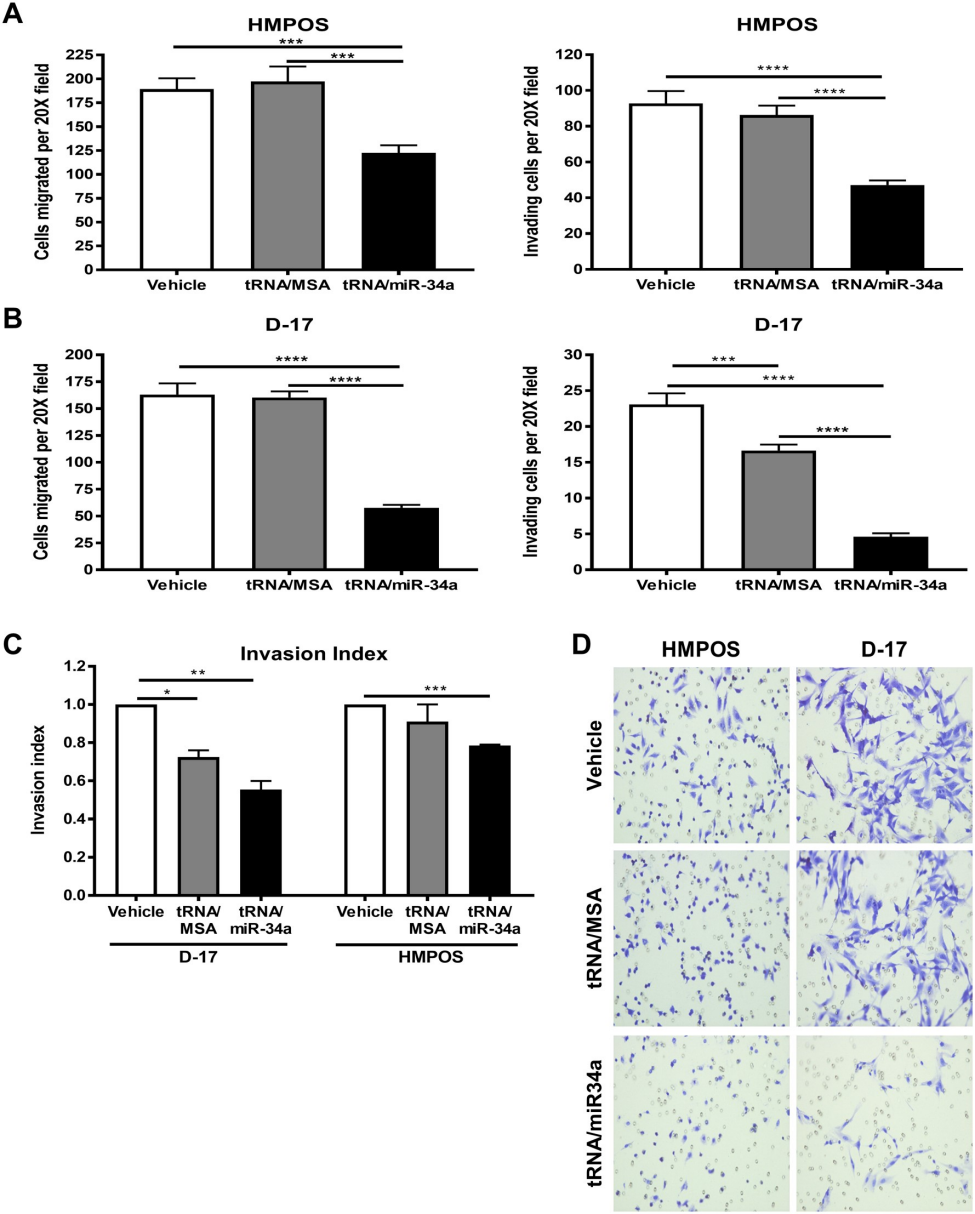
Cell migration and invasion was significantly inhibited in canine OSA cell lines transfected with tRNA/miR-34a prodrug. HMPOS (A) and D-17 (B) cells were treated with 5 nM tRNA/miR-34a or tRNA/MSA or vehicle for 48 hours and subjected to transwell invasion and migration assays for 24 hours, fixed with 95% ethanol:acetic acid and stained with crystal violet. Data are represented as mean±SEM. (C) Invasion index (mean±SEM) was calculated by dividing the % invasion of treated cells by the % invasion of vehicle cells. Data (A-C) were analyzed by one-way ANOVA multiple test followed by a Bonferroni test (*p <0.05, **p <0.01, ***p <0.001, ****p <0.0001). (D) Representative phase-contrast microscopy images (20X) of transwell migration assay.

We further determined whether tRNA/miR-34a prodrug alters the migratory behavior and invasive capability of HMPOS and D-17 cells, since both processes are critical for tumor metastasis and progression. Invasiveness and migration were significantly reduced in HMPOS (Fig 4A and 4D) and D17 (Fig 4B and 4D) cells treated with tRNA/miR-34a (P<0.001). While tRNA/MSA did not exert any effect on migration or invasion in HMPOS cells, this compound significantly reduced the invasion of D-17 cells (P<0.01). This was also reflected in the calculated invasion index (Fig 4C), which depicts the percentage of migrating cells that are also capable of invasion through the matrigel-coated membrane. These results demonstrate that the tRNA/mir-34a prodrug has the ability to reduce *in vitro* indicators of metastatic activities in canine OS cell lines.

### Genetically engineered tRNA/miR-34a prodrug is successfully processed in canine osteosarcoma cells and regulates the protein and gene expression of miR-34a targets

**Fig 5:**
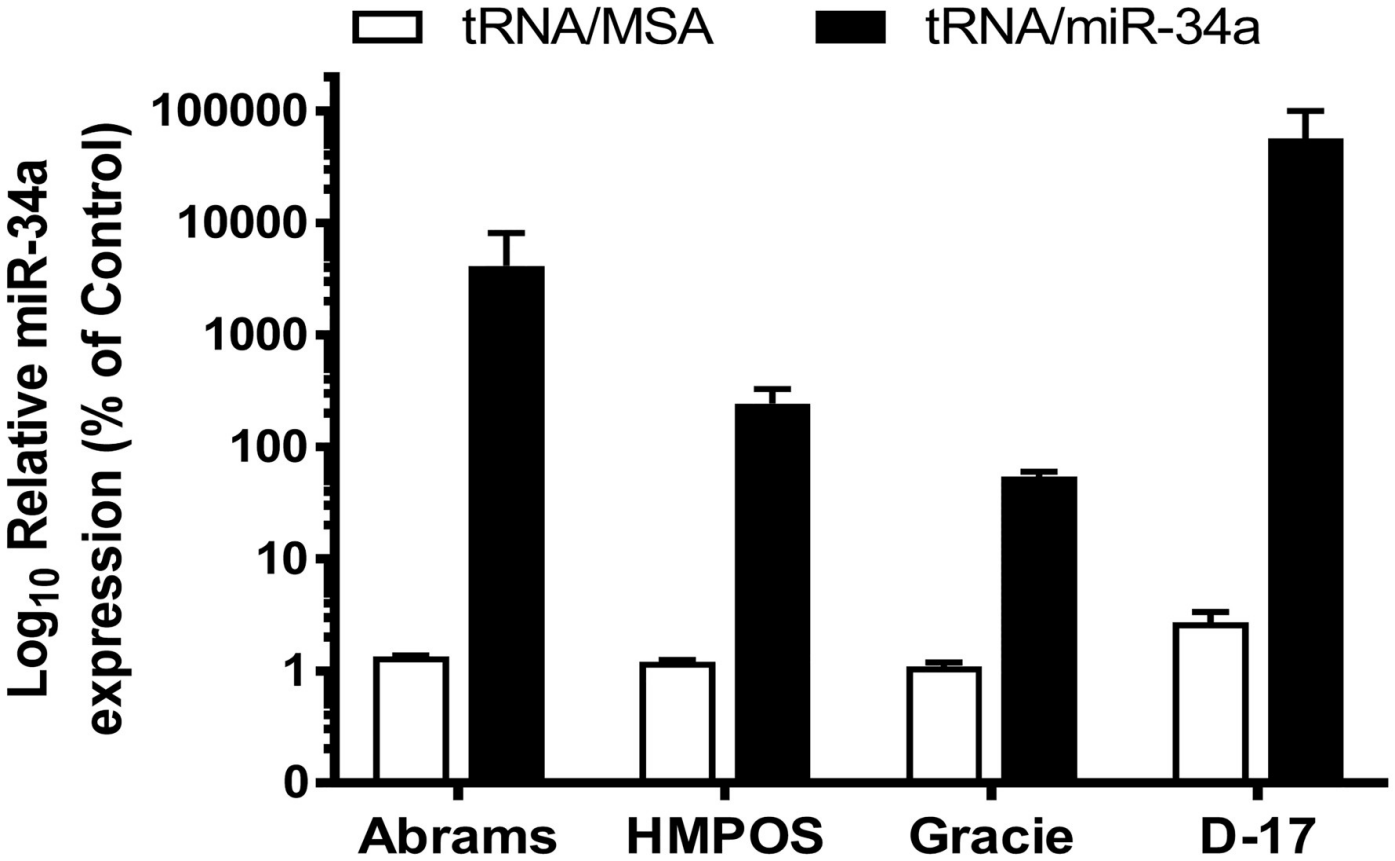
Bioengineered tRNA/miR-34a prodrug was processed to mature miR-34a in canine OS cell lines. Relative expression levels of mature miR-34a in cells 24 hours after treatment (10 nM tRNA/MSA or tRNA/miR-34a) were analyzed by qRT-PCR and normalized versus geometric mean of housekeeping genes *Rps5*, *Gapdh* and *Hnrnph1*. Data (mean±SEM) were analyzed independently analyzed by Student’s *t*-test.

**Fig 6:**
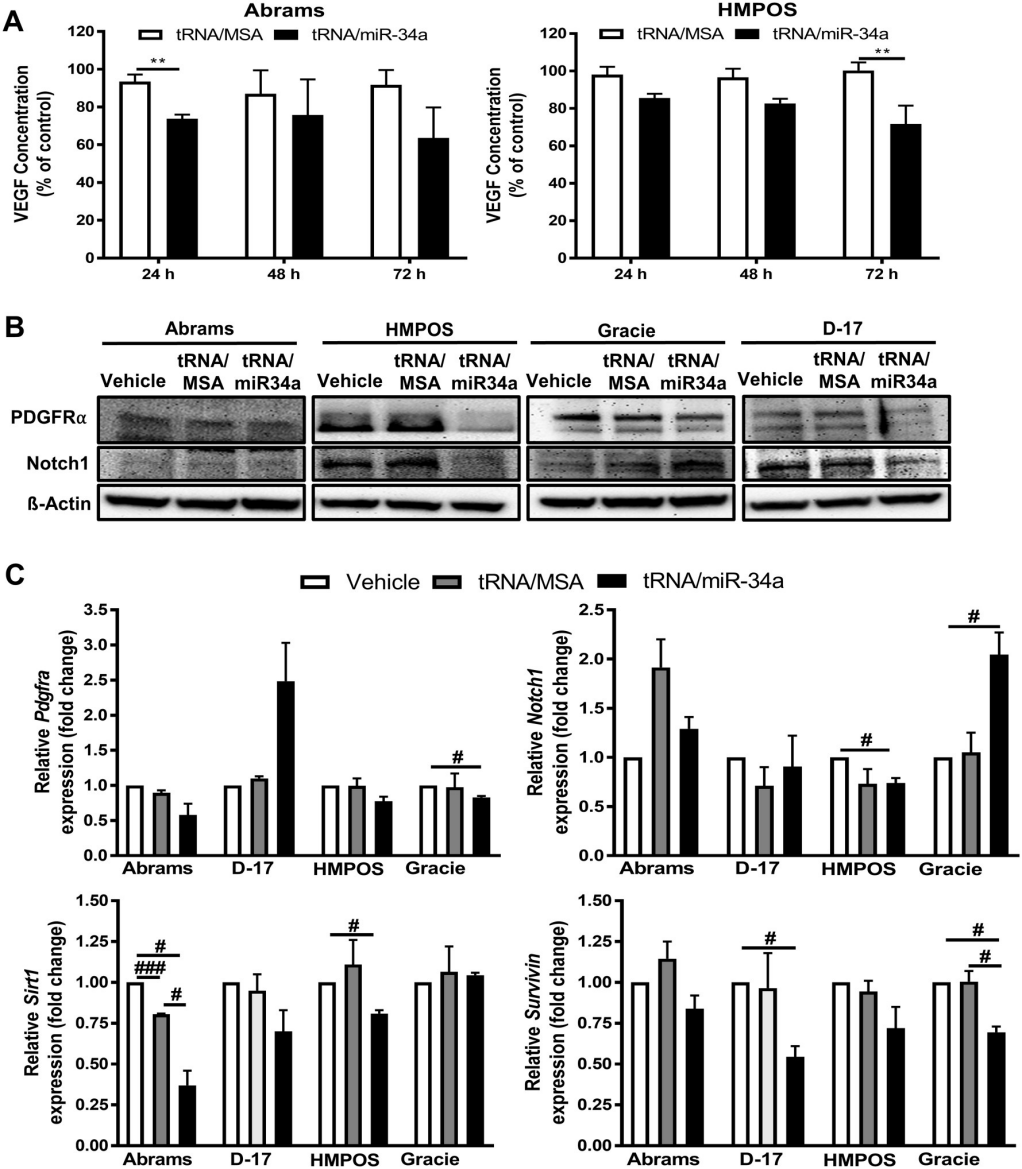
tRNA/miR-34a prodrug regulated both protein and gene expression of miR-34a targets in canine OS cell lines. (A) ELISA of VEGF released from cells after 24, 48 or 72 hours of treatment with 5 nM tRNA/MSA or tRNA/miR-34a. Data (mean±SEM) were calculated as percentage of control (vehicle) and analyzed by two-way ANOVA multiple test followed by a Bonferroni test (**p <0.01). (B) Representative western blot images of PDGFRα and Notch1, in cells treated with 10 nM tRNA/MSA or tRNA/miR-34a. ß-Actin was employed as loading control. (C) Relative mRNA expression levels of *Pdgfra*, *Notch1*, *Sirt1* and *Survivin* were analyzed by qRT-PCR and normalized versus geometric mean of housekeeping genes *Rps5*, *Gapdh* and *Hnrnph1*. Data (mean±SEM) were analyzed independently analyzed by Student’s *t*-test (#*p* <0.05, ###*p* <0.001).

To confirm that the cytotoxic effects and suppression of migration and invasion in canine OS cells by tRNA/miR-34a prodrug are associated with increased miR-34a levels, we measured mature miR-34a levels using microRNA-specific qRT-PCR method and examined expression levels of putative miR-34a target genes and proteins with qRT-PCR and western blot, respectively. Our results demonstrate a substantial increase in mature miR-34a levels in canine OS cells treated with tRNA/miR-34a prodrug. We identified that the magnitude of increase in mature miR-34a levels was cell line specific, with D-17 showing the highest fold change (56,854-fold) and Gracie the lowest fold change (54-fold) (Fig 5). We measured the amount of secreted VEGF in cell culture supernatants by ELISA in the HMPOS and Abrams cells and found that the tRNA/miR-34a caused a time-dependent reduction in secreted VEGF which was greatest at 72 hours (Fig 6A). In addition, western blot analysis revealed that HMPOS and D-17 cells treated with tRNA/miR-34a showed a decrease in PDGFRα and Notch1 protein levels after 48 hours of treatment. Interestingly, the protein expression of PDGFRα and Notch1 was not altered in Abrams at 48 hours post treatment. Furthermore, Gracie cells treated with tRNA/miR-34a prodrug showed an increased expression in Notch1 and only a slight decrease in PDGFRα (Fig 6B). We also looked at mRNA expression levels via qRT-PCR for several target genes of miR-34a including *Pdgfra*, *Notch1*, *Sirt1* and *Survivin* and found cell line dependent regulation of expression by tRNA/miR-34a (Fig 6C). The mRNA levels for *Pdgfra* were consistent with the findings from the western blot analysis with Abrams, HMPOS and Gracie having reduced levels while D-17 showed increased expression. Similarly, *Notch1* mRNA levels were increased in the Gracie cell line but reduced in the HMPOS cell line, mirroring the western blot results. We identified that all four cell lines have reduced expression of *Survivin* mRNA following tRNA/miR-34a treatment, with significant reductions seen in D-17 and Gracie (P<0.05). With regard to the relative *Sirt1* expression, only the Abrams and HMPOS cell lines demonstrated a significant reduction following tRNA/miR-34a treatment (P<0.05). These results demonstrate that the human tRNA/miR-34 a prodrug is successfully processed into a mature, functional miR-34a in canine OS cells and is capable of altering mRNA and protein levels of predicted targets.

### Bioengineered tRNA/miR-34a prodrug demonstrates tumor growth inhibition in a subcutaneous murine xenograft tumor model

**Fig 7:**
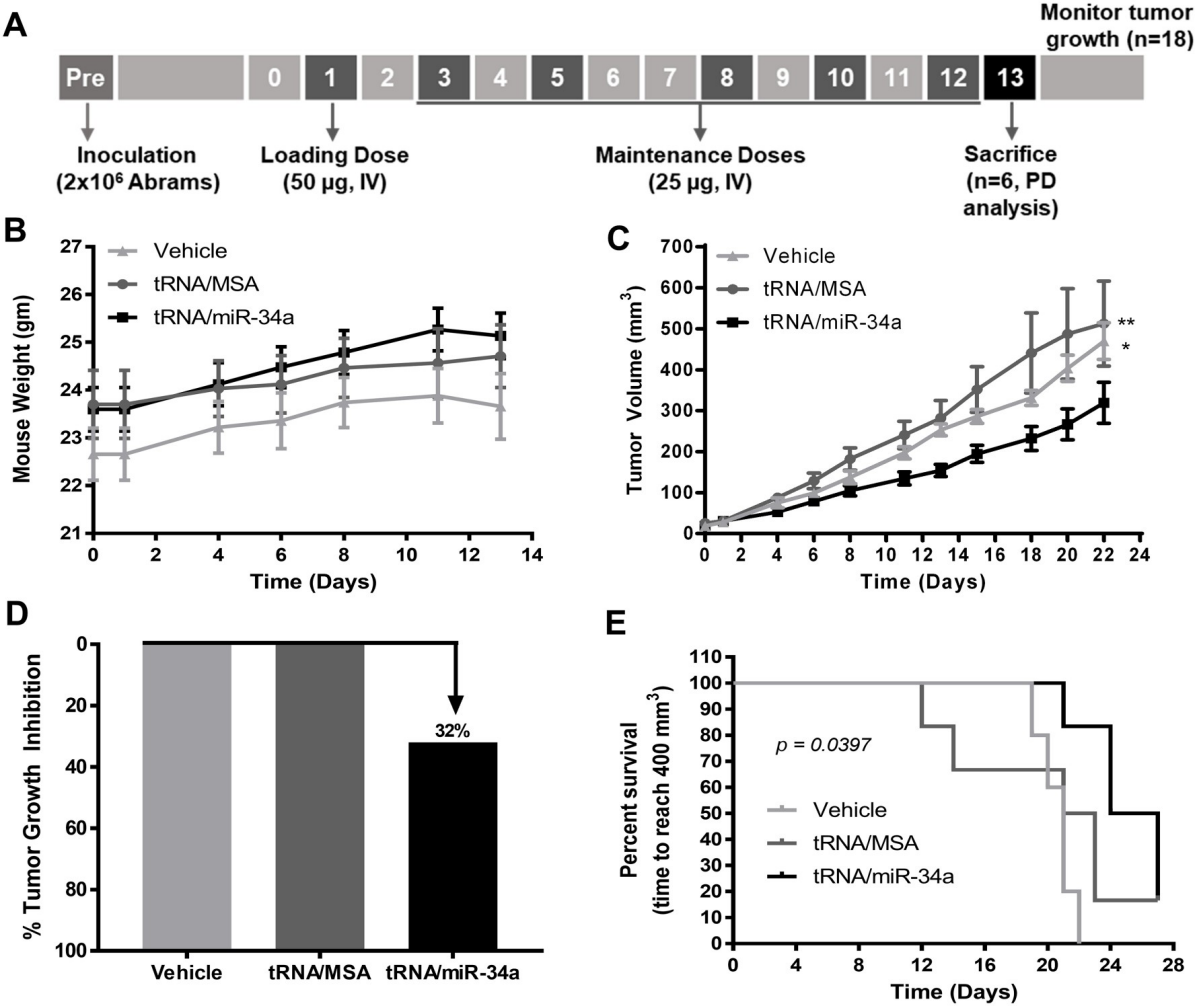
Tumor progression was suppressed by bioengineered tRNA/miR-34a prodrug in a subcutaneous murine xenograft tumor model. (A) Timeline of cell inoculation, drug administration and monitoring of tumor growth. For evaluation of early pharmacodynamics (PD) effects, three mice from the tRNA/miR-34a and three mice from the tRNA/MSA group were sacrificed 24 hours after last dose. (B) Monitoring of the mice weight during prodrug administration in each group. (C) Xenograft tumor growth in athymic nude mice treated with vehicle, tRNA/MSA or tRNA/miR-34a. Data (mean±SEM) were analyzed by repeated-measures two-way ANOVA followed by Bonferroni multiple-comparison test (*p <0.05. **p <0.01). (D) Tumor growth inhibition in mice treated with tRNA/MSA, tRNA/miR-34a or vehicle. (E) Survival curves, time for the xenograft tumor to reach 400 mm^3^, were generated by the Kaplan-Meier method and survival differences between groups compared using log-rank (Mantel-Cox) analysis.

**Fig 8:**
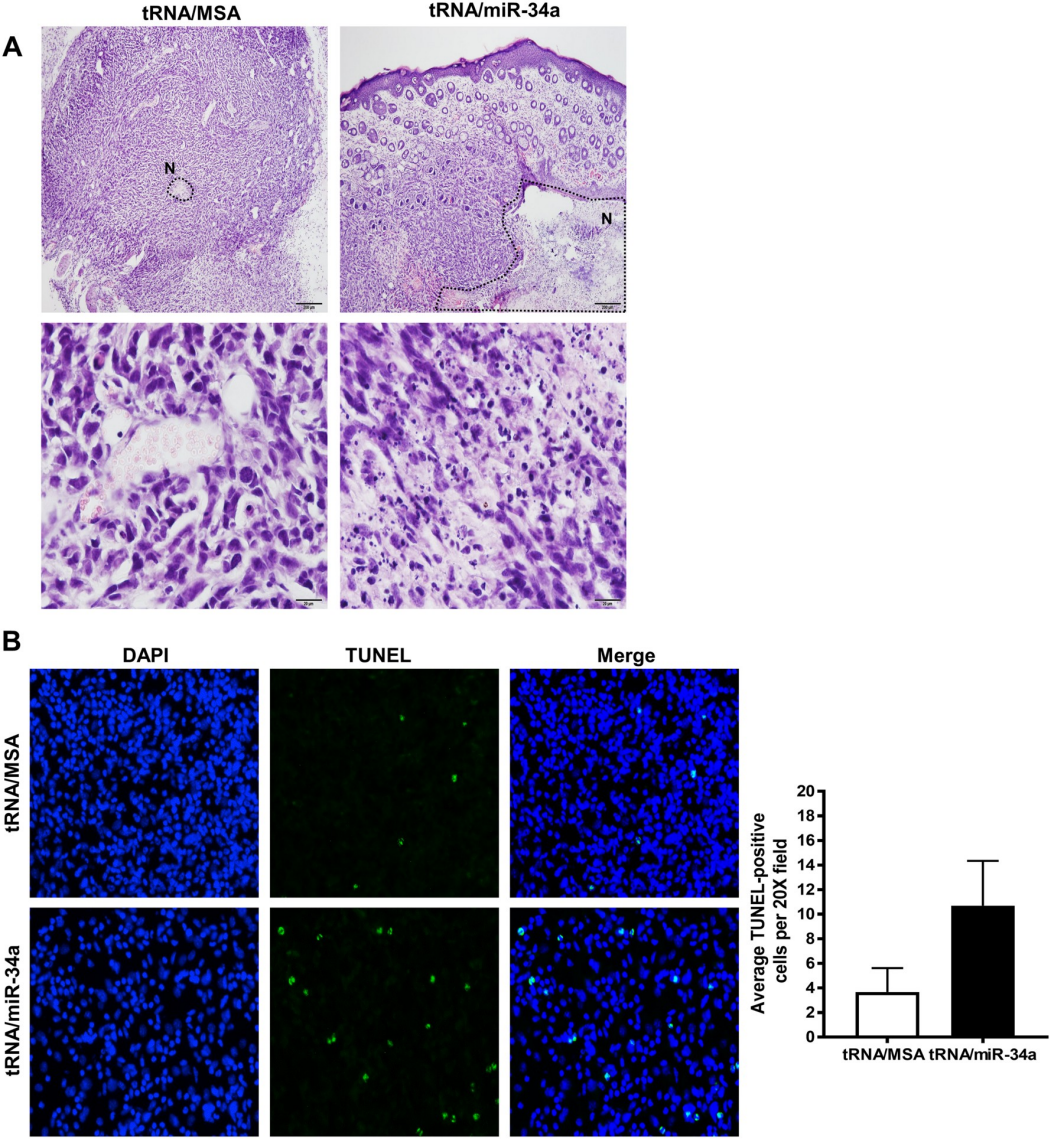
Bioengineered tRNA/miR-34a prodrug induced necrosis and infiltration by lymphocytes in the canine xenograft tumors. (A) Representative images of H&E staining from xenograft tumor section. Images show differences in degree of tumor necrosis (round dot line labeled with N; 10X magnification, top row) and infiltration by lymphocytes (20x magnification, bottom row). (B) Representative images of TUNEL staining from xenograft tumor section: DAPI (blue) and TUNEL (green), and summary of TUNEL-positive cells quantification per 20X field.

**Fig 9:**
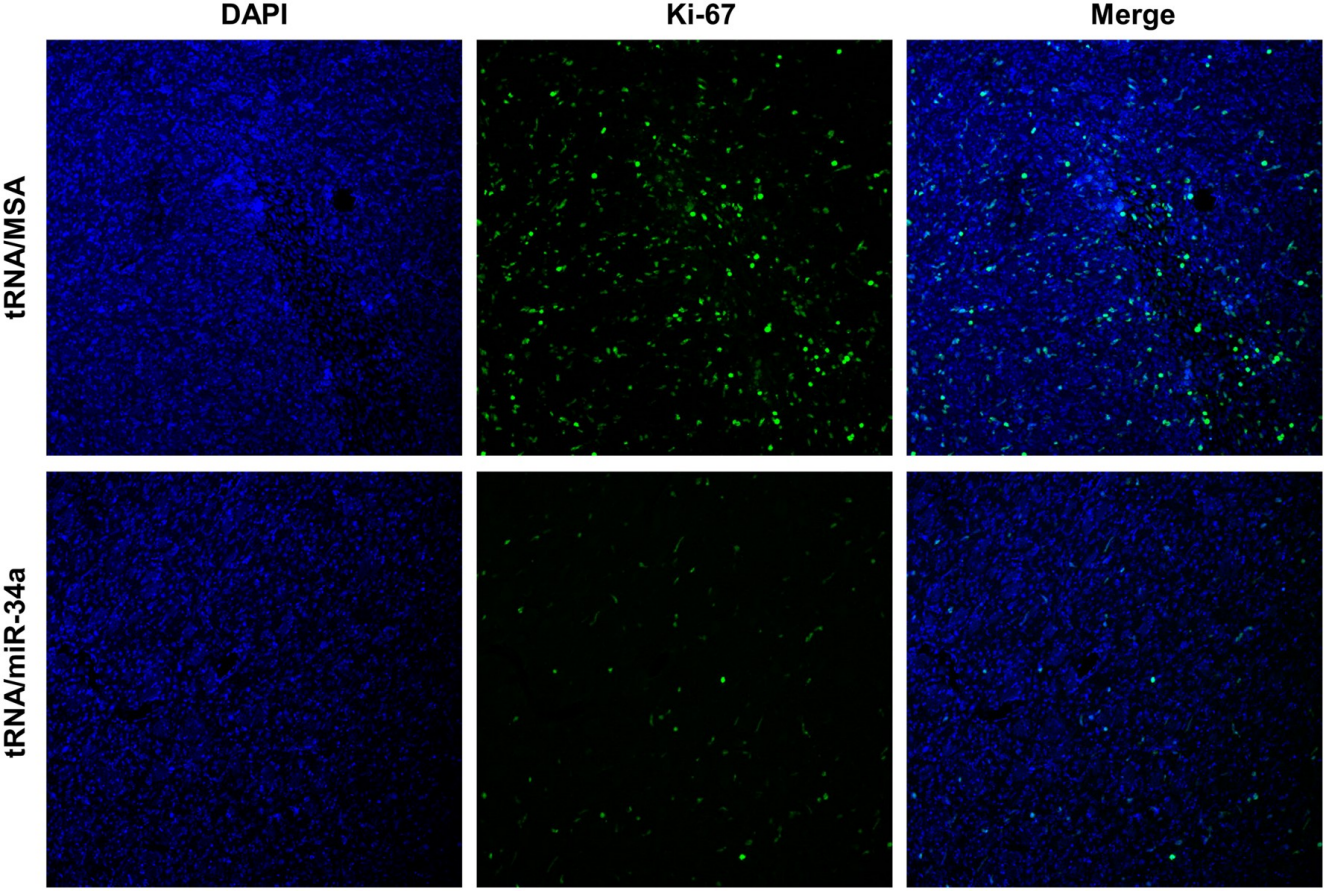
Genetically engineered tRNA/miR-34a prodrug compromised cellular proliferation in the canine xenograft tumors. Representative images of Ki-67/MIB-1 staining from xenograft tumor section: DAPI (blue) and Ki-67/MIB-1 (green) (10x magnification).

Based on the promising results that we obtained with *in vitro* investigations of the tRNA/miR-34a on canine OS cells, we next investigated the therapeutic effects of tRNA/miR-34a prodrug *in vivo* by employing a subcutaneous murine xenograft tumor model of the Abrams OS cell line. Mice bearing established tumors were treated with tRNA/miR-34a prodrug, tRNA/MSA or *in vivo-*jetPEI vehicle control by intravenous tail vein injection three times per week for six treatments. The treatment schedule is depicted in Fig 7A. None of the treatments had a negative effect on mouse weights (Fig 7B and S1 Fig), and the tRNA/miR-34a prodrug induced a significant reduction in tumor growth rate (P<0.01) and an overall 32% tumor growth inhibition compared to tRNA/MSA or vehicle (Fig 7C and 7D). This translated to a significant increase in survival when growth to a volume of 400 mm^3^ was used as the surrogate endpoint (Fig 7E; P = 0.0397). In the subset of tumors removed 24 hours following the final intravenous treatment, histological analysis by hematoxylin and eosin staining revealed a greater degree of tumor necrosis with infiltration of lymphocytes in mice treated with tRNA/miR-34a (Fig 8A). Increased apoptosis was also identified in TUNEL-stained tumor sections (Fig 8B) from the tRNA/miR-34a treated mice. Moreover, consistent with the reduction in tumor growth rate, xenograft tumors tissues from mice treated with tRNA/miR-34a showed lower nuclear Ki-67/MIB-1 labeling levels than mice treated with tRNA/MSA (Fig 9), demonstrating that this novel prodrug inhibits the proliferative ability of canine xenograft tumors.

## Discussion

Growing evidence has demonstrated that expression of microRNAs is found to be dysregulated in several diseases, such as hepatitis, cardiovascular diseases and cancer. While the use of miRNAs in the most current cancer clinical trials are as biomarkers for prognosis and drug efficacy, their ability to target numerous mRNAs that are altered in cancer makes these non-coding RNAs remarkable therapeutics targets [13, 14]. The expression of miR-34a is commonly lost or decreased in many types of human cancers such as leukemia, glioblastoma, breast carcinoma and even osteosarcoma (OSA) [44–46]. Importantly, miR-34a acts as tumor suppressor by regulating over 800 mRNAs involved in cellular plasticity, proliferation and survival, cell cycle, angiogenesis, or immune invasion in multiple cancer cells [22, 23]. Indeed, the re-expression of miR-34a levels in cell lines derived from diverse tumors, such as human and canine OSA, through administration of miR-34a-expressing vectors or synthetic miR-34a mimics has confirmed these anti-tumors effects associated with enhanced cell cycle arrest, apoptosis and suppression of metastatic abilities [19, 25–27, 45, 47]. Given that miR-34a expression is frequently decreased in cancer and its ability to regulate multiple oncogenic mRNAs, there is an increasing interest in developing novel therapeutic strategies to restore miR-34a levels.

The novel bioengineered miR-34a prodrug employed in this study is a natural and large molecule RNA agent designed by *Yu et al* [37]. This prodrug is selectively processed intracellularly to mature and functional miR-34a, in contrast to chemically synthetized miR-34a mimics that bear many artificial modifications that may alter structural properties, pharmacological and biological activities, and safety profiles [48]. The exploration of new therapeutic approaches also requires the use of relevant clinical models, and this has heightened importance in human OSA due to its low prevalence in the population. Unlike human OSA, canine OSA is highly prevalent, and diverse studies have demonstrated that canine and human OSA are genomically undistinguishable. Furthermore, OSA tumors from both species similarly express lower levels of miR-34a than non-malignant bone tissue [5, 6, 19].

Our data demonstrate that tRNA/miR-34a treatment results in a significant suppression of cellular viability in a concentration- and time-dependent manner in all canine OSA cell lines, but also reveal for the first time that the miR-34a prodrug can exert a long-term effect on cell proliferation as is shown by a meaningful reduction in clonogenic cell growth. Our data also suggested that this significant decrease in cell viability is due, in part, to enhanced apoptosis in canine OSA cells. These findings are in the line with previous reports showing a similar effect of tRNA/miR-34a prodrug in human OSA cells [30, 43] and lung and liver carcinoma cell lines [29]. Similarly, several studies carried out with synthetic miR-34a mimics and viral vector-based miR-34a expression systems demonstrated the negative impact of the enforced expression of miR-34a in cellular viability in human OSA [26, 47] and other tumor cells such as colorectal [44], glioblastoma [46] or endometrial [24] cancer cells. However, a recent study in canine OSA cells showed that re-introduction of miR-34a did not affect cellular proliferation [19]. This discrepancy might be explained by the differences in the methods employed to evaluate this parameter or to restore miR-34a levels. As seen in previous reports using lung, liver and bone cancer cells [29, 30] the transfer RNA scaffold (tRNA/MSA) is capable of suppressing cellular proliferation and colony formation, albeit to a lower degree and requiring higher concentrations. This anti-proliferative effect may be explained by formation of tRNA-derived RNA fragments (tRFs). Although tRFs can be constitutive components of cells, some are selectively produced under stressful conditions including nutrient deprivation, hypoxia or oxidative damage [49]. Recent findings have demonstrated that tRFs may play a dual role in tumorigenesis through regulation of invasion, cellular proliferation, metastasis, and gene expression [50]. Indeed, some tRFs inhibit cell proliferation and metastasis in cancer cells through diverse mechanism such as destabilization of YBP-1, inhibiting Notch signaling by targeting JAG2, or suppressing protein synthesis [51, 52]. The methionyl tRNA scaffold employed in the biosynthesis of miR-34a prodrug is susceptible to human RNases, and thus can be processed to tRFs which may exert an anti-proliferative effect at high doses by triggering apoptosis [29, 30] as is suggested by the increase in caspase-3/7 activity at 10 nM tRNA/MSA. However, it is noteworthy to point out that the lower doses of tRNA/MSA intravenously injected in our xenograft OSA tumor murine model did not affect tumor growth, consistent with the results reported previously in other xenograft tumor models [29, 30]. Our data also suggested that HMPOS cells (*p53* mutant) were the least sensitive to tRNA/miR-34a prodrug and D-17 cells (*p53* wild-type) the most sensitive, this may be related to differential p53 activities [31, 32] and/or differences in mRNA expression of the targets of miR-34a [19].

*Yu et al* showed that the restoration of miR-34a through bioengineered tRNA/miR-34a prodrug induces a suppression in migratory behavior and invasive capability of human OSA cells [30, 43]. Similarly, the ectopic expression of miR-34a by means of synthetic miR-34a mimics or viral vector systems also results in a decreased *in* vitro and/or *in* vivo metastatic ability of human [26, 47, 53] and canine [19] OSA cell lines. Consistent with these previous studies, in this study the migration and invasion of canine OSA cell lines were significantly inhibited following the treatment with tRNA/miR-34a. The significant suppression of invasiveness in D-17 cells by tRNA/MSA might be attributable to the production of tRFs. These cross-species data confirm the inhibitory effect of miR-34a prodrug in metastatic abilities in OSA cell lines.

Abrams OSA cells are commonly employed to produce xenograft tumors in murine models due to their aggressive phenotype, high degree of invasiveness and absence of growth inhibition by contact [54]. Concordant with previous reports of orthotopic and subcutaneous xenograft tumor models [29, 30, 43], the systemic administration of tRNA/miR-34a at therapeutic doses significantly suppressed the canine OSA xenograft tumor growth supported by an increased cellular apoptosis and tissue necrosis, and reduced cellular proliferation in comparison to tRNA/MSA and vehicle control. Furthermore, the treatment was well-tolerated by mice demonstrated by a lack of weight loss during the study, which is in line with previous studies showing a similar effect of tRNA/miR-34a prodrug in mice with absence of liver and kidney toxicity [30, 43]. Although tRNA/miR-34a had an anti-tumor effect in our xenograft tumor model, we did not observe neither significant increase in mature miR-34a levels nor decreased expression of its target genes in terminal tumor tissues (i.e. 24 hours after the last maintenance dose (S2 Fig)). These findings are consistent with studies recently published showing absence of miR-34a accumulation and target response after the last dose of tRNA/miR-34a in a full therapy study [29, 30, 43]. Therefore, taking into account that the time point selected was not optimal to evaluate these two parameters in the tumor tissues, an independent pharmacokinetic study should be performed to determine the timing of miR-34a changes in the tumor during a full therapeutic study.

The present study also demonstrated that the human bioengineered tRNA/miR-34a prodrug was successfully imported and selectively processed in canine OSA cells to high levels of mature miR-34a, consistent with previous results shown in human tumor cells [29, 30, 43]. Interestingly, the degree of increase in mature miR-34a levels was cell line specific, with D-17 exhibiting the highest increment. The p53 tumor suppressor protein regulates miR-34a expression, but miR-34a overexpression may also enhance p53 protein and down-regulate gene expression of multiple p53 transactivation inhibitors, such as *MDM4* and *SIRT1*, thus increasing p53 activity [55]. This strong interplay between miR-34a and p53 might explain why D-17, which carries a *p*53 wild-type [31] was the most sensitive to this miR-34a prodrug. Importantly, mature miR-34a derived from this novel prodrug was functionally active which is supported by the decreased protein and/or mRNA expression of putative miR-34a target genes including VEGFA, NOTCH1, SIRT1, PDGFRA and SURVIVIN, in the majority of the canine OSA cell lines. These miR-34a targets are involved in critical processes in cancer cells such as regulation of cell cycle and survival, migratory behavior and invasive capability, developmental of neovasculature and sensitivity to chemotherapeutic agents [24, 56–58]. Interestingly, we also identified cell-line dependent increased expression of some putative targets of miR-34a which may be explained by potential differences in the response to the stress of transfection as numerous stimuli (i.e. contact inhibition, serum starvation) have been identified to cause a switch from inhibition to activation of translation of some targets by miRNA [59]. Thus, the altered expression of aforementioned miR-34a targets provides a rational explanation for the negative effects of tRNA/miR-34a prodrug on cell viability and proliferation, clonogenic growth, cell survival, and metastatic abilities in canine OSA cells *in vitro*, as well as the inhibition of tumor growth and increased necrosis in the subcutaneous canine OSA xenograft tumor model in athymic mice.

## Conclusions

Our findings demonstrated that the novel bioengineered humanized tRNA/miR-34a prodrug is successfully processed in canine OSA cells, increasing mature miR-34a levels and subsequently producing anti-tumor effects by regulating the expression of miR-34a target oncogenes. Moreover, systemic administration of tRNA/mir-34a prodrug in canine OSA xenograft tumors in murine models was well tolerated at therapeutic doses, and inhibited the tumor growth. Therefore, our data support the concept of using canine OSA as a useful model for informing future clinical development of this novel therapeutic agent.

## Supporting information

S1 Fig Monitoring of the xenograft tumor growth. Monitoring of chronological changes of the tumor growth in each mice.
(TIF)

S2 Fig Evaluation of both miR-34a and mRNA of its targets genes in the xenograft tumors. Relative expression levels of miR-34a (A) and mRNA of its target genes (B) were were analyzed in tumor tissues 24 hours after the 6th dose (14 days since first injection) by qRT-PCR and normalized versus geometric mean of housekeeping genes *Rps5*, *Gapdh* and *Hnrnph1*. Data (mean±SEM) were analyzed independently analcyzed by Student’s *t*-test.
(TIF)
