# Supplementary figures and images for: A genetically engineered microRNA-34a prodrug demonstrates anti-tumor activity in a canine model of osteosarcoma
Source: PLoS One. 2018 Dec 31;13(12):e0209941. doi: 10.1371/journal.pone.0209941 (PMC6312226; doi:10.1371/journal.pone.0209941)

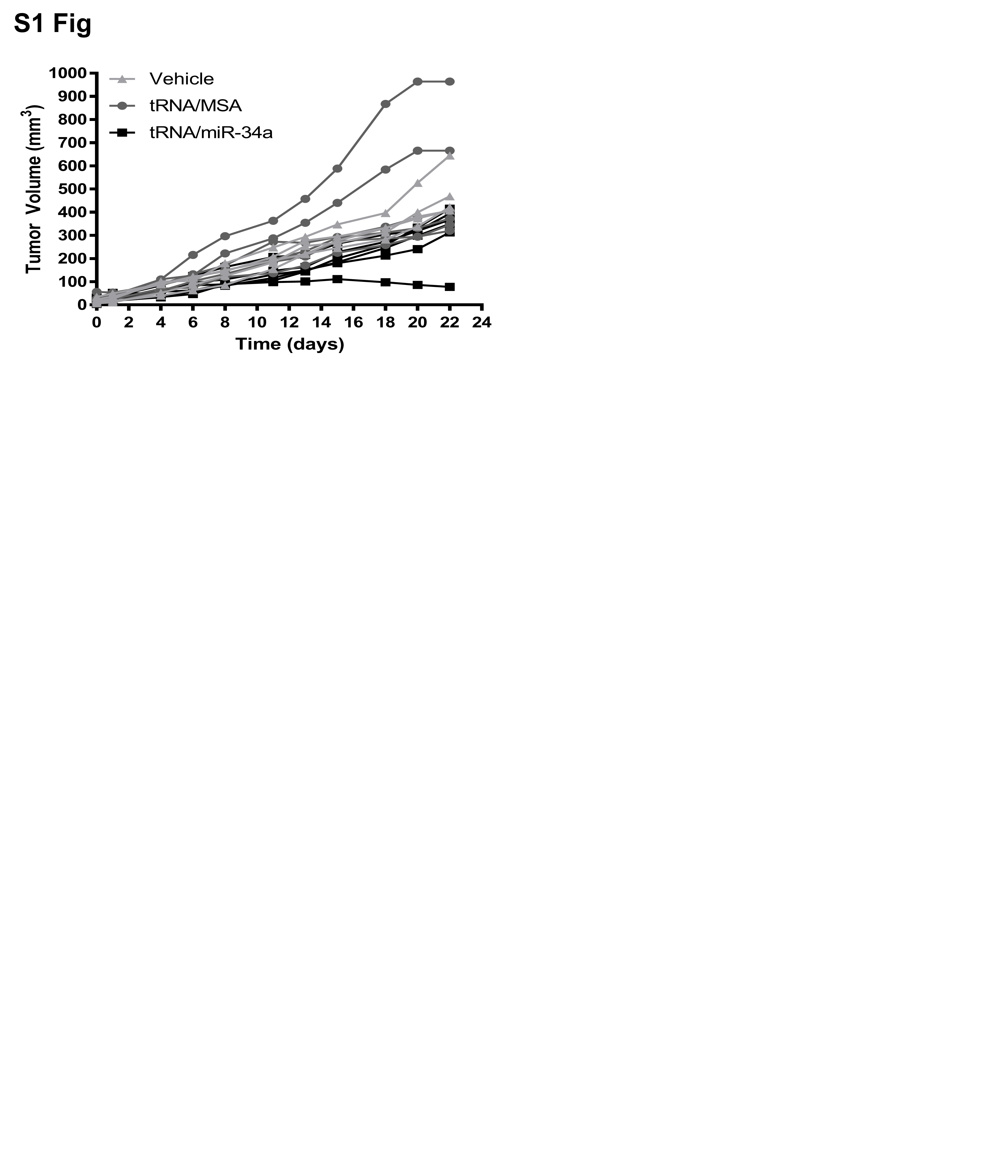

Supplement: S1 Fig — Monitoring of chronological changes of the tumor growth in each mice. (TIF) [file pone.0209941.s001.TIF]

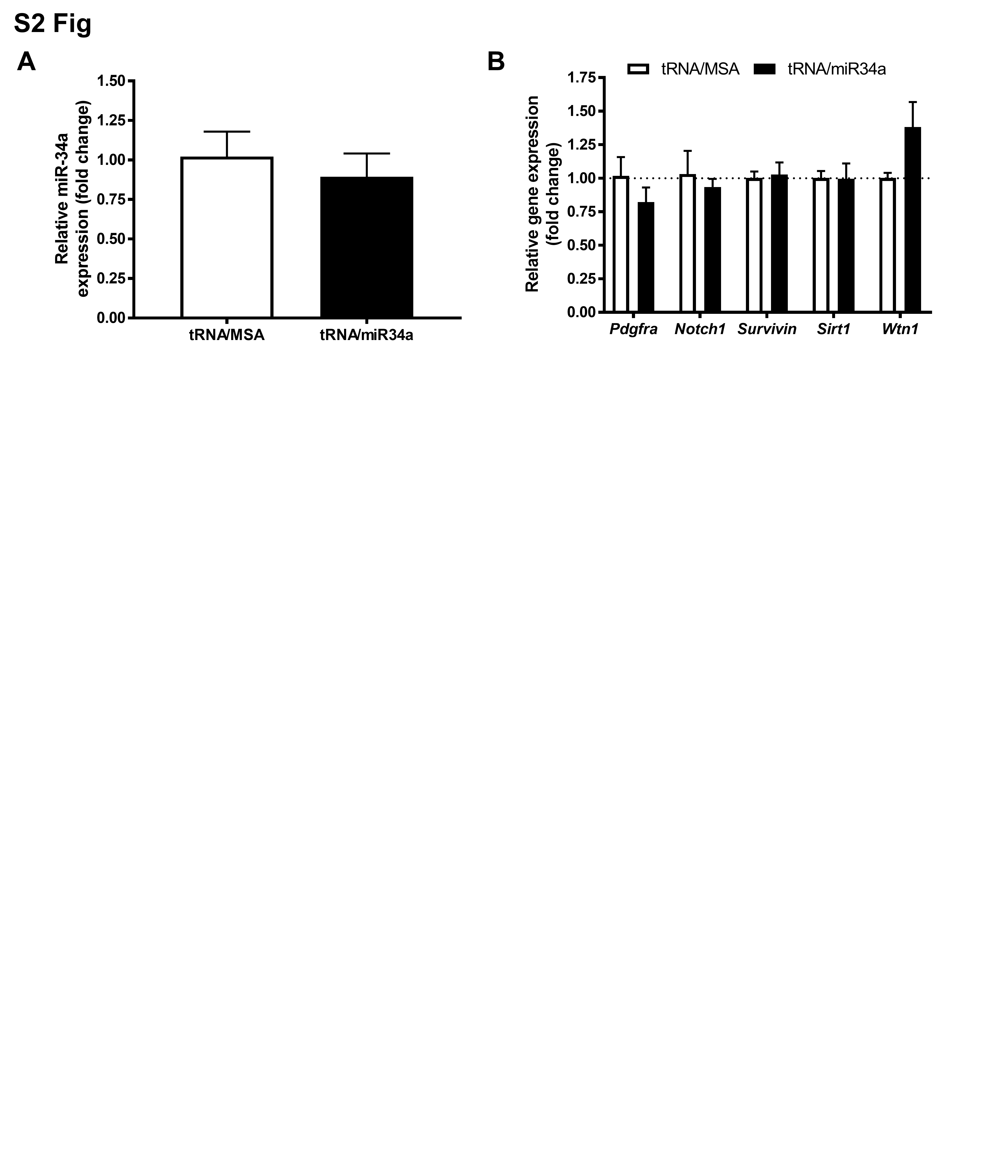

Supplement: S2 Fig — Relative expression levels of miR-34a (A) and mRNA of its target genes (B) were were analyzed in tumor tissues 24 hours after the 6th dose (14 days since first injection) by qRT-PCR and normalized versus geometric mean of housekeeping genes Rps5, Gapdh and Hnrnph1. Data (mean±SEM) were analyzed independently analcyzed by Student’s t-test. (TIF) [file pone.0209941.s002.TIF]
